# One-Year Prospective Association of BMI with Later Cognitive Development in Preschoolers

**DOI:** 10.3390/brainsci12030320

**Published:** 2022-02-27

**Authors:** Carina Hansen, Latasha Smith, Brian A. Lynch, Antonela Miccoli, Magdalena Romanowicz, Loren Toussaint

**Affiliations:** 1 Department of Psychology, Luther College, Decorah, IA 52101, USA; carina.k.hansen@dmu.edu; 2 Department of Science and Math, Central Baptist College, Conway, AR 70532, USA; lsmith@cbc.edu; 3 Department of Pediatric and Adolescent Medicine, Mayo Clinic, Rochester, MN 55905, USA; lynch.brian@mayo.edu (B.A.L.); antonelamiccoli@gmail.com (A.M.); 4 Department of Psychology and Psychiatry, Mayo Clinic, Rochester, MN 55905, USA; romanowicz.magdalena@mayo.edu

**Keywords:** obesity, child development, preschool child

## Abstract

This study examined the prospective relationships between preschoolers’ body mass index (BMI) and cognitive development. BMI, cognitive (i.e., Brigance), sex, and age data were collected from seven cohorts of children attending Head Start from 2012 to 2018. Children (*N* = 324) with two years of complete data were included. After controlling for the first year cognitive development scores, age, gender, and the cohort, the BMI was predictive of lower cognitive development scores in year two (*B* = −0.06, β = −0.14, *t* = −3.19, *p* = 0.002). Female sex (*B* = 2.69, β = 0.10, *t* = 2.30, *p* = 0.022) and older age (*B* = 0.02, β = 0.15, *t* = 3.20, *p* ≤ 0.001) were also shown to be statistically significant predictors of improved year two cognitive scores. The initial BMI scores were associated with poorer one year cognitive development scores in this sample of preschool children. Excessive body mass may contribute to numerous biological, psychological, and social factors that inhibit children with obesity from reaching their full cognitive potential, during a time in which brain development and cognitive skills development are at critical points of growth. Early childhood obesity interventions may have positive consequences for cognitive development, but additional prospective studies are needed to confirm these results.

## 1. Introduction

Obesity is an epidemic in the United States [1]. As of 2018, the obesity prevalence level had increased to 19.2% in children aged 2–19 years [2]. Children aged 2 to 5 years have an obesity prevalence of 13.4%, going up to 20.3% in those aged 6 to 11 years [3]. In children, being overweight is considered as between the 85th and 95th percentile compared to children of the same age and sex, while being over the 95th percentile is considered obese [4]. Obesity in preschool children is important because nearly 90% of children who are obese at age 3 years are overweight or obese later in life [5], and similarly, children who were overweight at age 5 years showed a ten times greater risk of obesity later in life [6]. Hence, the early years of a child’s life are crucial and can impact being overweight and obesity later in life [7,8]. These trends in obesity among children, and the positive association with subsequent obesity in adulthood, are a growing health concern.

Research suggests that there are numerous physical health problems linked with obesity and being overweight, including the increased risk of diabetes, fertility difficulties, cardiovascular problems, obstructive sleep apnea, asthma, impaired immune function, and cancer [9,10,11]. Commonly observed in adults, these obesity- and overweight-related health risks are appearing with increasing prevalence in obese and overweight children [12]. In addition to these, there have been recent findings of an increased risk of upper extremity physeal fractures in children classified as overweight [13]. Studies have also found associations between higher BMI and the increased risk of elevated blood pressure [14].

Although much is known about the physical health consequences for obese and overweight children and adolescents, much less is known about the cognitive consequences. One of the first studies conducted on the connection between obesity and cognition in children showed a correlation of higher BMI to lower cognitive development in adolescents [15]. This has been speculated to be due to inflammation of the brain [16], or changes in the prefrontal cortex [17]. Another study looked at the link between visceral obesity and age-related cognitive decline and suggested pro-inflammatory cytokines or insulin resistance to be the possible mechanism [18]. A recent study showed that children aged 43–84 months who were overweight, had lower cognitive performance than those with a healthy weight, on a nonverbal intelligence test that focused on logical reasoning [19]. Similarly, a more recent study reported better scores on the Bayley-III development test (standard developmental test for infants) in healthy participants than those who were overweight [20]. In Mexico, 33 participants between six and eight years old demonstrated similar results, showing that overweight children performed worse than normal-weight children on measures of verbal fluidity [21].

Interestingly, research on cognition and the body mass index (BMI), studied as a continuous variable instead of the BMI classification (obesity, overweight, and normal weight), also shows differences in cognitive performance among adolescents. In one study [15], the authors reported that adolescents aged eight to sixteen years old, with higher BMIs, perform poorer on block design cognitive assessments than those with lower BMIs. Another study found that participants aged six to sixteen years old, that had an increase in their BMI over a year, had lower comprehension scores [22]. This was also confirmed in two studies on obese adolescents which found that they performed poorly on non-verbal IQ tests and had deficits in cognitive functioning [23,24].

Although there is strong evidence that elevated or increasing BMI can have a negative impact on cognitive capabilities in children over 5 years of age [15,22,24], there is a gap in the knowledge regarding how this impacts cognitive capabilities in preschool children. Not only can high BMI start early in life, but the first few years of a child’s life are crucial to brain development and building a foundation for future learning [25]. For this reason, more research must be conducted using younger children.

The purpose of the present study was to examine a northeast Iowa Head Start population, with seven cohorts assessed between 2012 and 2018, and to examine how the initial BMI percentile scores were associated with cognitive development scores one year later, controlling for the initial cognitive development scores. To examine this, seven cohorts of children defined by their year of entry into Head Start, had data collected from the beginning of their first year to the beginning of their second year in Head Start (two years are the maximum allowable years in Head Start). Improvement in cognitive development was expected for all the children as they continued to age and participate in the Head Start curriculum. It was expected that there would be differences in the amount of improvement in cognitive development depending on a child’s initial BMI percentile. Controlling for a child’s cognitive level at the beginning of the first year, it was expected that differences in cognitive levels in the second year would be explained using the first year BMI percentile scores. In short, we hypothesized that those with a higher initial BMI percentile would experience less improvement in cognitive development in year two.

## 2. Materials and Methods

### 2.1. Participants

Data were collected from preschool children enrolled in fourteen different Head Start locations in 7 different northeast Iowa counties. There were 1065 preschoolers (aged 2 to 5 years) attending these Head Start locations from 2012 to 2018, and data were gathered for all of them. No exclusions for race/ethnicity, sex/gender, or underlying medical conditions were made. Of the 1065 children attending during the study years, 455 participated for two years. Of these 455 preschoolers, 227 (49.9%) were female, while 228 were male (50.1%). Of the 455 preschoolers, 18 (4%) of them were in Cohort 1, 43 (9.5%) were in Cohort 2, 62 (13.6%) were in Cohort 3, 92 (20.2%) were in Cohort 4, 82 (18%) were in Cohort 5, 78 (17.1%) were in Cohort 6, and 80 (17.6%) were in Cohort 7. Of the participants, 17 were 2 years of age (3.7%) at the start of their first year in Head Start, while 395 (86.8%) were 3 years of age. There were 43 (9.5%) participants who were missing data for how old they were at the beginning of their time in Head Start. The participants consisted of 39 (8.6%) from Cresco, 33 (7.3%) from Decorah, 7 from Elkader (1.5%), 33 (7.3%) from Guttenberg, 29 (6.4%) from Monona, 32 (7.0%) from New Hampton, 100 (22.0%) from Olewein, 69 (15.2%) from Postville, 57 (12.5%) from Waukon, 28 (6.2%) from Waverly, and 28 (6.2%) from West Union. The children spent a minimum of one year and a maximum of two years at Head Start. There were several different reasons why some children only participated in this program for one year, one of them being that children who were four years or older were prioritized to ensure that they were ready for kindergarten, as this is one of the main focuses of Head Start. In addition to this, transportation to Head Start was not provided for children under the age of four years, making it impossible for some children to participate. Sometimes, parents chose to have their children not enroll until they were four years or older, even though they qualified for an earlier enrollment. Lastly, some children who completed the first year of Head Start did not return to complete the second year.

As the data was all de-identified, we do not have socio-demographic statistics specific to the participants who had qualifying data from years one and two in the program. Rather, we have statistics for everyone enrolled in Head Start in northeastern Iowa in 2018. Of the children enrolled in Head Start, 85% were aged three to four years old. There were 228 families enrolled in Head Start in 2018. Of these, 117 (51%) were single-parent families with 63% of them being employed. The other 111 (49%) were two-parent families, with 50% of parents both employed, 41% with only one parent employed, and 9% with both unemployed. All the families had federal assistance provided for them, most of them from the Supplemental Nutrition Assistance Program and Special Supplemental Nutrition Program for Women, Infants, and Children. Race/ethnicity varied with almost 70% Caucasian, 10% biracial, 4% African American and 16% “other”. The primary language was English in 90% and Spanish in 9% of families. (Personal communication with Sharon Burke, sburke@neicac.org, Early Childhood Program Director, March 2020). This study was found to be exempt by the Institutional Review Board under 45 CFR 46.101 (b) (4) [26]. The Human Ethics Approval Number was 2019_14.

### 2.2. Measurements

#### 2.2.1. Cognitive Development

Brigance Screens III is a nationally standardized and validated developmental screening tool for children from infancy to first grade [27]. It includes a Brigance Head Start that aligns with the domains of the Early Learning Outcomes Framework and evaluates children on predictors of school success [28]. In accordance with federal requirements, this assessment is taken by a child within 45 days of entering Head Start [29]. The test has a maximum score of 100 that can be achieved and is administered in 10 to 15 min. The total score is then compared to age-appropriate cutoff scores that detect probable giftedness or areas of delay in a child. The Brigance includes assessments across 3 domains; for children aged 2 years and older, these are language development, motor skills, academic and cognitive skills.

#### 2.2.2. Body Mass Index

Height and weight assessments were taken for the participants by Head Start (HS) staff and Luther College nursing students, using digital scales and stadiometers. The BMI percentile was then calculated for the students using ChildPlus software [30].

#### 2.2.3. Demographic Controls

Child sex, age, Head Start site/classroom, and cohort year were available in our retrospective de-identified data.

### 2.3. Procedures

Brigance was administered by HS staff to the children in the fall, within the first 45 days of starting Head Start. BMI and cognitive measures were recorded between July and December in each cohort year. For all the children included in the study, follow-up BMI and cognitive data were collected between July and December of the child’s second year at HS. Those who did not have two years of complete data were excluded from the study. Retrospective data collection was carried out by the research assistant (CH), supervised by HS staff onsite, and included: (1) the Head Start site location, (2) dates in Head Start, (3) sex, (4) date and age (in days) for every height and weight assessment, (5) height and weight, and (6) the Brigance score. Data were entered into a password-protected Excel sheet and then de-identified before analysis.

### 2.4. Analyses

Statistical Package for the Social Science version 25 was used to conduct statistical analyses. Multiple regression analyses were conducted to see if there was an association between year one BMI and year two cognitive development, controlling for year one cognitive development, cohort, site, sex, and age (the most parsimonious model that still allowed for the examination of the study hypothesis). Assumptions of linear multiple regression modeling were tested and met. There was no collinearity (VIFs < 1.2). Linear relationships between the predictors and outcome were confirmed (via a scatterplot). Residuals were normally distributed (confirmed by a histogram plot), homoscedastic (confirmed by a residual by outcome plot), and independent (Durbin–Watson = 1.66). No transformations were applied to any of the data. Statistical significance was set at *p* < 0.05.

## 3. Results

Of the 1065 preschool students who had data collected between 2012 and 2018 in northeast Iowa Head Start, a total of 324 of them had two years of data for body mass index, Brigance assessments, age, and sex, and were included in this study. After controlling for the first year cognitive development score, the initial BMI percentile was predictive of less cognitive development in year two (*B* = −0.06, β = −0.13, *t* = −2.90, *p* = 0.004). To further rule out this being due to any other variables, we included gender, age, and cohort, and found the statistically significant association of initial BMI percentile with cognitive development after a year remained relatively unaffected (*B* = −0.06, β = −0.14, *t* = −3.19, *p* = 0.002) (see Table 1). In addition to the initial BMI percentile having an association with cognitive development scores after a year, female sex (*B* = 2.69, β = 0.10, *t* = 2.30, *p* = 0.022) and older age (*B* = 0.02, β = 0.15, *t* = 3.20, *p* ≤ 0.001) were also shown to be statistically significant predictors of year two cognitive development scores. The child’s cohort year was not found to be a statistically significant predictor of improved cognitive development at year two: Cohort 1 (*B* = 11.25, β = 0.07, *t* = 1.52, *p* = 0.129), Cohort 2 (*B* = 3.81, β = 0.07, *t* = 1.44, *p* = 0.152), Cohort 3 (*B* = 2.35, β = 0.07, *t* = 1.21, *p* = 0.228), Cohort 4 (*B* = 1.64, β = 0.05, *t* = 0.92, *p* = 0.359), Cohort 5 (*B* = 1.68, β = 0.05, *t* = 0.89, *p* = 0.372), and Cohort 6 (*B* = 3.50, β = 0.10, *t* = 1.86, *p* = 0.064). Two exploratory analyses confirmed that the prospective association of BMI with cognitive development was consistent for both boys and girls (*B* = 0.00, β = −0.08, *t* = −0.88, *p* = 0.381) and for both younger and older children (*B* = −0.39, β = −0.25, *t* = −0.79, *p* = 0.432).

## 4. Discussion

The aim of this study was to explore what association the initial BMI percentile would have with a preschooler’s cognitive development. As expected, those with a higher initial BMI saw less improvement between year one and year two in their cognitive development. These findings are supported by current research from the NHANES data for adolescents, aged eight to sixteen years [15,31], however, this was found for two to five year olds in the present study. Older age was shown to have a statistically significant association with higher cognitive development scores after one year. This could be attributed to known improvements in inhibition as a child ages, allowing them to better engage in classroom learning [32]. Age was a significant predictor of cognitive development, similar to what has been found in previous studies [33]. In a previous study with 6634 individuals, 8–21 years old, age was found to have a significant effect on cognitive development, with effect sizes ranging from Beta = 0.10 to 0.16 [33]. In the present study, a similar-sized effect was found with three to five year olds, showing an association of age with cognitive development (Beta = 0.15). Perhaps most significantly, there was an equivalent effect size to that of age for the association of BMI with cognitive development (Beta = −0.14). One possible reason for this finding is that obesity may cause changes in brain function, in areas such as the prefrontal cortex, which can then suppress cognitive development and function [17]. Additionally, there is evidence that obesity causes inflammation in the brain, which may lead to metabolic disturbances that could be associated with cognitive impairment [16]. Further research is needed to determine the biological mechanisms of the association of BMI with decreased cognitive development.

The current study suggests that, as early as two years of age, BMI could be having an impact on the immediate subsequent cognitive abilities and learning in children. Although BMI was shown to be a part of the prediction of cognitive development just one year later, there could be longer implications. For instance, if this deleterious association persisted every year through a child’s senior year of high school, one could see this having significant implications for future cognitive abilities and grades [34,35]. As intelligence has been reported as a strong long-term predictor of job performance, this can affect someone’s future career [36].

In addition to finding evidence to support our hypothesis, we also found evidence that females had higher cognitive development scores after a year than males. One explanation that has been offered is that females at a young age are better able to regulate their attention, a key to learning [37]. Although sex and age were significant predictors of cognitive development, there was no evidence to support the notion that a child participating in a different cohort year of the program had any association with the change in their cognitive development scores.

A key strength of this study is the prospective one-year design and analysis. Another strength is that our data come from multiple years. Additionally, this research uses a very young age group that has not been thoroughly studied. Last, this study used a validated measure of cognitive development that is not used in the present literature on this topic. The limitations of this study include a racially and ethnically non-diverse sample, the use of one dependent variable, and that the participants only had two years of data. A key limitation of our single outcome analysis is that the total Brigance score (which is all we could access) encompasses language development, motor skills, academic and cognitive skills. The inclusion of motor skills in the total score may present a confound to the measurement of cognitive development per se, and to its association with obesity, as motor skills may be affected by obesity (e.g., limiting the range of motion, bodily mechanics, etc.) in ways that are not mediated by the brain. Future research could evaluate specific areas of cognitive development to determine if BMI is differentially associated with specific aspects of cognition, such as memory, language, visuo-spatial ability, perception, attention, and executive functions. For example, in one study BMI was found to be related to visuo-spatial ability but not with memory [31]. Additionally, data were de-identified, preventing us from collecting household demographics for each child, and data came to us in aggregate form, limiting our ability to examine separate dimensions of cognitive development and test the internal consistency of the Brigance assessment. Another limitation is that different tools were used to measure weight and height throughout this study; ideally the measuring tool would be kept consistent. The limited data on how body mass index affects cognitive development in preschoolers still makes this a useful study, but more research will need to be conducted to apply it to populations outside of Head Start, and to see if the association continues at older ages.

## 5. Conclusions

In summary, a higher initial BMI percentile in preschoolers is associated with less cognitive development one year later, with an effect size equivalent to that of age. Further research needs to be conducted to understand the exact biopsychosocial mechanisms of this association. Furthermore, research should be carried out to see if this applies to more racially and geographically diverse populations.

## Figures and Tables

**Table 1:** The Multiple Regression Coefficients for BMI Percentile Prospectively Predicting Cognitive Development One Year Later.

Variable	*B*	β	*t*	*p*
Brigance Y1	0.38	0.56	12.47	0.001 *
BMI	−0.06	−0.14	−3.19	0.002
Gender	2.69	0.10	2.30	0.022
Age	0.02	0.15	3.20	0.001
Cohort 1	11.25	0.07	1.52	0.129
Cohort 2	3.81	0.07	1.44	0.152
Cohort 3	2.35	0.07	1.21	0.228
Cohort 4	1.64	0.05	0.92	0.359
Cohort 5	1.68	0.05	0.89	0.372
Cohort 6	3.50	0.10	1.86	0.064

* *p* ≤ 0.001.

## Data Availability

Data will be made available upon request.
